# Supplementary material for: Complexome profiling on the Chlamydomonas lpa2 mutant reveals insights into PSII biogenesis and new PSII associated proteins
Source: J Exp Bot. 2021 Aug 26;73(1):245–62. doi: 10.1093/jxb/erab390 (PMC8730698; doi:10.1093/jxb/erab390)
Supplement: erab390_suppl_Supplementary_Dataset_S1 [file erab390_suppl_supplementary_dataset_s1.zip › Supplemental Dataset 1 - Excel List and all profiles/ProteinDistances.html]

### Interactive chart of migration distances

(left) zNormed intensities of wild type and mutant.  
(right) Adjusted euclidean distance of each protein pair.  
  
Note: Distance is 36 if signal of WT or Mut is missing. Click and zoom vertically for more details
